# Health Facility Graduation from Donor-Supported Intensive Technical Assistance and Associated Factors in Zambia

**DOI:** 10.1371/journal.pone.0131084

**Published:** 2015-06-22

**Authors:** Phillip Koni, Nathaniel Chishinga, Lameck Nyirenda, Prisca Kasonde, Richard Nsakanya, Michael Welsh

**Affiliations:** 1 FHI 360, PO Box 320303, Lusaka, Zambia; 2 Cardno, PO Box 320303, Lusaka, Zambia; University of Missouri-Kansas City, UNITED STATES

## Abstract

**Introduction:**

The FHI360-led Zambia Prevention Care and Treatment partnership II (ZPCT II) with funding from United States Agency for International Development, supports the Zambian Ministry of Health in scaling up HIV/AIDS services. To improve the quality of HIV/AIDS services, ZPCT II provides technical assistance until desired standards are met and districts are weaned-off intensive technical support, a process referred to as district graduation. This study describes the graduation process and determines performance domains associated with district graduation.

**Methods:**

Data were collected from 275 health facilities in 39 districts in 5 provinces of Zambia between 2008 and 2012. Performance in technical capacity, commodity management, data management and human resources domains were assessed in the following services areas: HIV counselling and testing and prevention of mother to child transmission, antiretroviral therapy/clinical care, pharmacy and laboratory. The overall mean percentage score was calculated by obtaining the mean of mean percentage scores for the four domains. Logistic regression models were used to obtain odds ratios (OR) and 95% confidence intervals (CI) for the domain mean percentage scores in graduated versus non-graduated districts; according to rural-urban, and province strata.

**Results:**

24 districts out of 39 graduated from intensive donor supported technical assistance while 15 districts did not graduate. The overall mean percentage score for all four domains was statistically significantly higher in graduated than non-graduated districts (93.2% versus 91.2%, OR = 1.34, 95%CI:1.20–1.49); including rural settings (92.4% versus 89.4%, OR = 1.43,95%CI:1.24–1.65). The mean percentage score in human resource domain was statistically significantly higher in graduated than non-graduated districts (93.6% versus 71.6%, OR = 5.81, 95%CI: 4.29–7.86) and in both rural and urban settings.

**Conclusions:**

QA/QI tools can be used to assess performance at health facilities and determine readiness for district graduation. Human resources management domain was found to be an important factor associated with district graduation.

## Introduction

The global agenda for HIV includes universal access to HIV prevention and treatment [1]. To achieve this, Zambia, like other resource-limited countries, relies on donor support. Zambia has benefited from the US President’s Emergency Plan for AIDS Relief (PEPFAR) and the Global Fund to Fight AIDS, Tuberculosis and Malaria (GFATM) in scaling-up HIV care and treatment services nationwide. However, it is recognized that a major challenge facing efforts to scale up HIV/AIDS services in Zambia and elsewhere in sub-Saharan Africa is the sustainability of services should donor support be removed or reduced.

The Zambia Prevention, Care and Treatment Partnership II (ZPCT II), a PEPFAR funded project through the United States Agency for International Development (USAID), supports the Government of Zambia in scaling up HIV and AIDS services in 5 provinces of Zambia. At the time of this study in 2012, a total of 350 health facilities (341 public and 9 private) were supported through this partnership, in implementing HIV/AIDS services. In 2005, the Zambian Ministry of Health (MOH) adopted a performance improvement approach (PIA) as a critical component in driving the improvement of quality of health care. “[PIA] is a step-by-step methodology for finding out what is needed to ensure good performance and delivering it; it uses quality tools in a guided and logical manner to attain performance targets and improve quality” [2].

To ensure the sustainability of HIV/AIDS services in these ZPCT II-supported health facilities, the ZPCT I (later on called ZPCT II in 2009) project introduced a quality assurance and quality improvement (QA/QI) system in 2007 [3]. The QA/QI data collected provided feedback to strengthen service provision, develop local capacity and improve overall monitoring and evaluation of different technical areas of HIV/AIDS services. The QA/QI system supported the provision of high quality HIV services, consistent with the National HIV Guidelines and Standards in all ZPCT II-supported health facilities. Importantly, the approach guided a tailored reduction in intensive technical support from ZPCT II, and transferred the bulk of the technical assistance responsibilities to the MOH district and provincial offices once the standards were reached and maintained: a process referred to as district graduation.

After graduation, the MOH and the Ministry of Community Development, Mother and Child Health (MCDMCH) through the Provincial Medical Office (PMO) and the District Medical Office (DMO) respectively, took responsibility of ensuring that there was provision of quality HIV/AIDS services through continued mentorship and monitoring of quality through the PIA approach in all government health facilities. This allowed ZPCT II to shift its focus to non-graduated districts and only conducted quarterly monitoring of the quality of services in the graduated districts.

Support to HIV services in Africa has been essential and lifesaving but countries including Zambia are now preparing to have less or flat lined donor support even as demand for services increases [4]. Over time, more of the financial burden for sustaining these services will be shifted to host countries. Strategies should be built into any project design and implementation to facilitate continuation of services beyond the period of external donor support [4]. Sustainability can be defined as the “capacity to maintain program services at a level that will provide ongoing prevention and treatment of a health problem” beyond external donor financial, managerial and technical support [5].

Elements of sustainability include technical, programmatic, social and financial elements. All four elements need to be addressed to achieve operational sustainability [6]. Other elements of sustainability include factors such as availability of drugs and equipment, staff establishment and continuing education and the overall working conditions in a facility. When these elements are missing the quality of services are impacted [5,7].

The concept of sustainability can be operationalized by grouping determining factors into three clusters: context (environmental factors), activity profile (type of services offered or activities carried out) and organizational capacity (capability to carry out set tasks faced by the organization) [4].These factors interact in different ways; the identification of gaps in these factors illuminates barriers to sustainability of services [4,7]. Torpey et al, in their case study concluded that achieving operational sustainability was possible in a resource limited setting through institutionalization of a QA/QI system. The authors illustrated how the use of a QA/QI system can make it possible to identify performance strengths and weaknesses in service delivery that are integral to operationalizing sustainability. They demonstrated how feasible and practical it is to prepare districts for graduation from project support by starting with improving the quality of services at individual health facilities and building up the districts' ability to manage the HIV services [4,7].

In this study we use the same concept of determining operational sustainability using a QA/QI system. We assess the QA/QI system that was used in the ZPCT II project to determine district graduation from intensive donor support, describe the graduation process and determine performance domains associated with district graduation.

## Methods

### Study setting and design

This was a retrospective review of data collected between July 2008 and December 2012 for QA/QI activities conducted in 275 of 350 (79%) ZPCT II-supported health facilities located in 39 of 42 targeted districts for graduation in five provinces. In this context, a district is a geographical and administrative area defined by government characterised by a cluster of health facilities. The health facilities within the districts were classified according to residence as either rural or urban facilities. The five provinces were Central, Copperbelt, Luapula, Northern and North Western Provinces. The process of district graduation assessment was rolled out in phases. At the time of this study, the rolling out of the assessment had not yet reached all the 42 districts; that is why only 39 districts were assessed. The 275 of the 350 health facilities belonged to the 39 assessed districts while the 75 remaining health facilities belonged to the three districts that were not yet assessed for graduation.

The QA/QI tool was administered on a quarterly basis to health facility staff who were working in the following four technical areas: HIV counselling and testing and prevention of mother to child transmission (CT-PMTCT), antiretroviral therapy/clinical care (ART-CC), Pharmacy and Laboratory. The QA/QI tool was designed to assess four quality performance domains: technical capacity, commodity management, data management and human resources management.

### Sample size and sampling strategy

There were 23 health facilities from 7 districts included that were supported during the ZPCT I period (2008–2009) and an additional 252 facilities from 32 districts included that were supported by ZPCT II (2009–2012). The process of assessing graduation started toward the end of ZPCT I, and facilities that were assessed in ZPCT I were not re-assessed in ZPCT II, the results discussed in this manuscript are therefore based on combined data for 275 facilities from 39 districts assessed for graduation in both ZPCT I and ZPCT II.

### District graduation process

A district with health facilities achieving and maintaining good quality services in line with existing MOH and Health Professions Council of Zambia national standards across all technical areas is targeted for graduation. The technical areas considered in the QA/QI tool were adherent to government guidelines for: (i) HIV counselling and testing and Prevention of Mother to Child Transmission of HIV services (CT-PMTCT), (ii) antiretroviral therapy and clinical care services (ART-CC), (iii) Laboratory services, and (iv) Pharmacy services. District graduation occurs when health facilities within a district score 90% and above in all the technical areas and maintain this good quality service for more than two consecutive quarters. The criteria was used for districts with five or more facilities supported by ZPCT II is that a minimum of 80% of health facilities in a district should score 90% and above. For districts with less than five ZPCT II-supported facilities, 100% of the health facilities should meet the criteria for graduation for the district to be graduated.

Graduation of a district does not mean elimination of financial support but rather reduced technical support. Facilities and districts that graduated received less technical support from ZPCT II as this responsibility of technical support was passed on to the MOH at district and provincial level as part of their ongoing monitoring system. The transfer of responsibility was done following planning and involvement from both the MOH and ZPCT II. However, a process of less intensive monitoring of service quality by ZPCT II continued after a district graduated in order to facilitate and assure sustained good quality of HIV services in line with national standards. During the post-graduation period, a comprehensive post-graduation management plan for each graduated district was developed that enabled quality service to be sustained within the local district health systems. This occurred through establishing a comprehensive system for continually monitoring the quality of care in each technical area of a facility in a graduated district and incorporating this into the standard MOH national QI system, that was implemented by the district health office team.

### QA/QI Tool and Graduation domains

The QA/QI tool contained four domains: technical capacity, commodity management, data management and human resource management (Table 1). In each domain, there are specific questions that are tailored toward the four technical service areas offered at the health facility (CT-PMTCT, ART-CC, Laboratory and Pharmacy). The options to the responses to each question in the QA/QI tool were dichotomous variables.

**Table 1:** Performance Domains by technical service area.

	Service Area			
Performance Domain	CT-PMTCT	ART-CC	LABORATORY	PHARMACY
**Technical Capacity**	Yes	Yes	Yes	Yes
**Commodity management**	Yes	Not applicable^1^	Yes	Yes
**Data management**	Yes	Yes	Yes	Yes
**Human Resources**	Yes	Yes	Yes	Yes

^1^ Commodity management under ART-CC is not independently assessed but is measured under Pharmacy service area; Yes, assessed.

#### Technical capacity

Facilities eligible for graduation must have incorporated technical strategies at all levels of service delivery and be able to consistently provide quality service as defined by the minimum national MOH standards.

#### Commodity management

Commodity management measured continuous availability of HIV test kits and chemical reagents for laboratory tests. Moreover, facilities must have been able to effectively forecast, quantify, order, procure and store ARVs, OI drugs, and other drugs including laboratory supplies under optimal conditions in order to eliminate stock outs and reduce overstocking of commodities.

#### Data management

This domain measured appropriate management of health information system which is critical for monitoring patient outcomes, planning and tracking progress and performance of programs. Quality of data collection, entry, generation and submission of reports across all technical areas was also assessed under this domain. Furthermore, correct completion of CT-PMTCT registers, ART clinic information and timeliness of report submission was measured as part of key criteria required to graduate. In addition, this domain assessed general management and use of electronic patient record management system.

#### Human resources management

The human resources management domain assessed availability of trained staff. In order to attain graduation status, facilities must had at least two trained health care workers providing services in various technical areas. This would ensure that staff transfers, staff changes and retirements would not disrupt service provision in the short to medium term.

### Ethical approval

Ethical approval was obtained from the FHI360 Protection Human Subjects Committee, North Carolina, USA and the ERES Converge Ethics Committee, Lusaka, Zambia.

### Data collection and operationalization

Data were extracted from a paper based QA/QI tool and entered into a Microsoft Access (Microsoft Corp., Redmond, USA) database for data management and exported to Microsoft Excel, Statistical Package for Social Sciences software version 21(SPSS Inc., Chicago, USA) and STATA version 12 (Stata Corp., College Station, Texas, USA) for analysis.

### Data analysis

We scored the type of technical service available in the health facilities (CT-PMTCT, ART-CC, Pharmacy, and Laboratory services) in each of the four domains. The total score for the responses for each technical area was obtained by summing up the individual responses to the questions in that technical areas. This total score was then divided by the maximum score for that technical area and then multiplied by 100 percent to obtain a percentage score of performance. The mean percentage score for each domain was then calculated by obtaining the mean of the percentage scores for the four technical areas in that domain. The overall mean percentage score for all four domains was then calculated by adding these mean percentage scores for the four domains and then dividing the sum by four to obtain a mean of mean percentage scores.

The outcome of interest was whether or not a district graduated following assessment using the QA/QI tool. We used logistic regression models to estimate the odds ratios (OR) and 95% confidence intervals (CI) of factors associated with district graduation. In addition, the mean percentage scores, OR and 95% CI of factors associated with district graduation were stratified by rural and urban facilities and by province using logistic regression models.

## Results

### Characteristics of the health facilities

Between 2008 and 2012, there were 39 districts with 275 health facilities that were assessed for district graduation. Of these health facilities, 110 (40%) were classified as urban and the remaining 165 (60%) as rural. Out of the 275 health facilities, 241 (87%) were primary health care facilities; 24 (9%) first-level referral hospitals; 7 (3%) second-level referral hospitals and 3 (1%) tertiary-level referral hospitals. All the 275 health facilities had CT-PMTCT services; 124 (45%) had ART-CC services; 103 (37%) Laboratory services and 124 (45%) Pharmacy services (Table 2).

**Table 2:** Characteristics of the 275 health facilities.

Area of residence		N = 275 n (%)
**Type of facility**	Urban	110 (40.0)
	Rural	165 (60.0)
**Services available**	Primary health care	241 (87.6)
	First-level referral hospital	24 (8.7)
	Second-level referral hospital	7 (2.5)
	Third-level referral hospital	3 (1.1)

**Fig 1:**
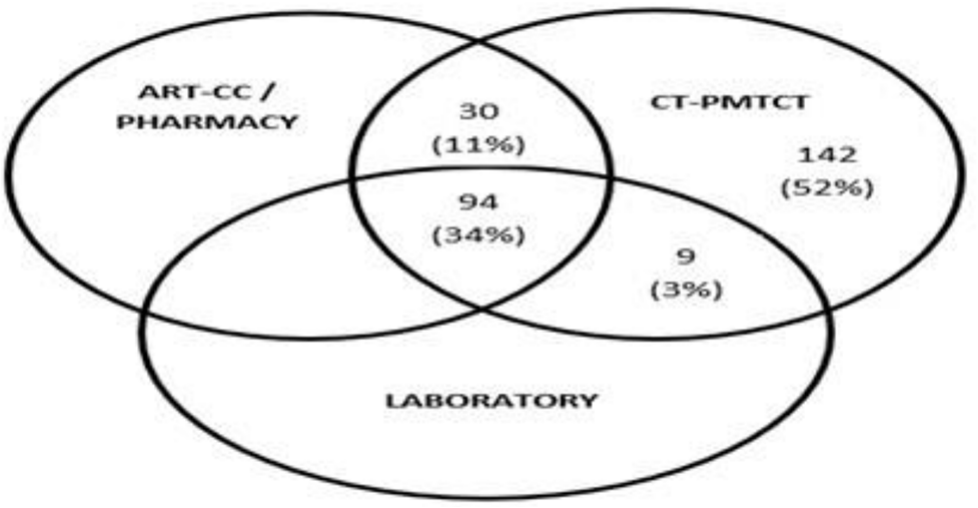
Venn diagram showing the distribution of technical services in the 275 health facilities. All health facilities that had ART-CC also had Pharmacy services. That is why ART-CC and Pharmacy are combined. Some health facilities had stand-alone CT-PMTCT services (n = 142). ART-CC, Pharmacy and Laboratory were not stand-alone.

Not all health facilities had all the four technical service areas (CT-PMTCT, ART-CC, Laboratory and Pharmacy) in the same locality. Out of the 275 health facilities, 94 (34%) had all four service areas in the same locality (Fig 1). The assessment for graduation in each health facility was therefore done according to what service area was available.

**Fig 2:**
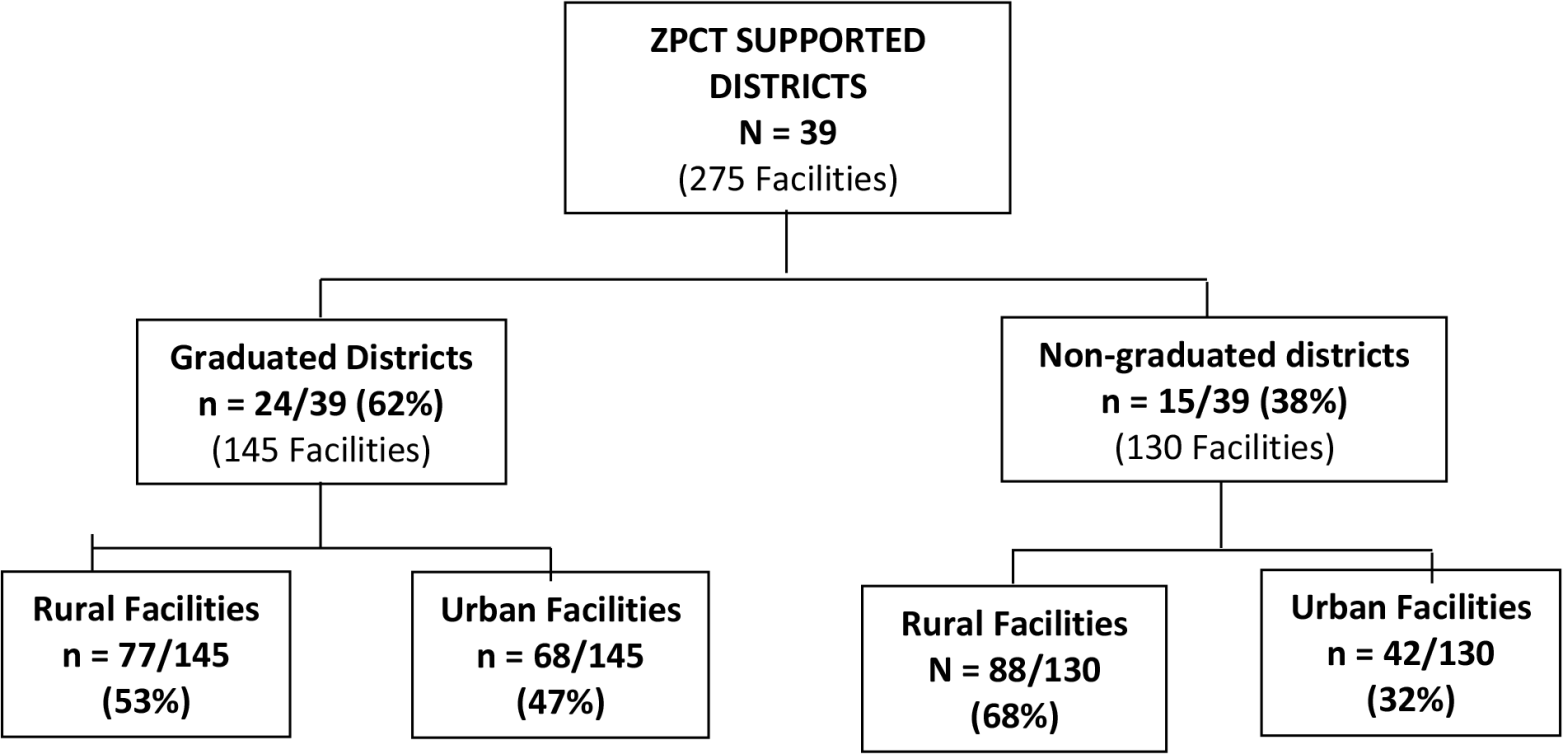
District Graduation performance.

Of the 39 districts (with 275 health facilities) assessed for graduation, 24 districts (62%) graduated from intensive donor supported technical assistance and 15 districts (38%) did not. The 24 districts that graduated had 145 health facilities while the 15 districts that did not graduated had 130 health facilities. In the districts that graduated, 77 (53%) out of 145 health facilities were rural based while the remaining 68 (47%) were urban. In districts that did not graduate, 88 (68%) out of the 130 health facilities were rural while the remaining 42 (32%) were urban. The proportion of rural facilities was less in districts that graduated than those that did not graduate; 53% versus 68%, respectively (Fig 2).

### Graduation Performance

The overall mean percentage score for all 4 domains was statistically significantly higher in graduated than non-graduated districts; 93.2% versus 91.2% (OR = 1.34, 95% CI: 1.20–1.49, P <0.001), respectively (Table 3).

**Table 3:** District Graduation Performance in four Performance Domains.

Domain	Program Area	Graduated % Score	Non-graduated % Score	OR (95% CI) for the Mean Scores	P-Value
**Commodity Management**					
	CT-PMTCT	96.0%	96.8%		
	Laboratory	93.1%	94.9%		
	Pharmacy	90.9%	92.5%		
**Mean percentage score**		**93.3%**	**94.5%**	**0.81 (0.63–1.05)**	**0.109**
**Data Management**					
	ART-CC	93.3%	92.9%		
	CT-PMTCT	98.0%	100.0%		
	Laboratory	97.1%	95.5%		
	Pharmacy	96.7%	92.0%		
**Mean percentage score**		**97.0%**	**97.4%**	**0.86 (0.56–1.31)**	**0.494**
**Human Resources**					
	ART-CC	94.5%	92.8%		
	CT-PMTCT	99.2%	81.7%		
	Laboratory	87.6%	81.7%		
	Pharmacy	91.6%	53.4%		
**Mean percentage score**	** **	**93.6%**	**71.6%**	**5.81 (4.29–7.86)**	**<0.001**
**Technical Capacity**					
	ART-CC	91.6%	93.0%		
	CT-PMTCT	93.8%	93.8%		
	Laboratory	87.6%	86.5%		
	Pharmacy	89.5%	86.4%		
**Mean percentage score**		**91.8%**	**91.3%**	**1.07 (0.92–1.23)**	**0.382**
**Overall mean percentage score**		**93.2%**	**91.2%**	**1.34 (1.20–1.49)**	**<0.001**

The mean percentage score in human resource domain was statistically significantly higher in graduated than non-graduated districts; 93.6% versus 71.6% (OR = 5.81, 95% CI: 4.29–7.86, P <0.001), respectively (Table 3). The human resources domain had the lowest mean percentage score of 71.6% in non-graduated districts compared to all other domains within non-graduated districts. Within Human resource domain, in the non-graduated districts, ART-CC service had the highest score of 92.8%, followed by CT-PMTCT and Laboratory scored 81.7% each and least score was in Pharmacy with 53.4%. In graduated districts, the least human resource domain performance score was in Laboratory with 87.6% (Table 3).

In the technical capacity domain, CT-PMTCT within graduated districts had the highest mean percentage scores (93.8%) followed by ART-CC service (91.6%), then Pharmacy (89.5%) and Laboratory (87.6%) with mean percentage scores below the graduation threshold of 90%. The graduation scores in the districts that did not-graduate followed a similar pattern as those that graduated;, with CT-PMTCT and ART-CC service areas scoring above the graduation threshold (93.8% and 93.0%, respectively) while Pharmacy and Laboratory had mean percentage scores below the graduation threshold (86.4% and 86.5%, respectively).

In commodity management domain there was little variation in mean percentage scores between graduated and non-graduated districts across CT-PMTCT, Pharmacy and Laboratory. The mean percentage scores in commodity management domain were above the graduation threshold in both graduated and non-graduated districts.

In data management domain, the mean percentage scores for ART-CC, CT-PMTCT, Laboratory and Pharmacy were high in both graduated and non-graduated districts. The domain mean percentage scores for data management in both graduated and non-graduated districts were 97% and 97.4% respectively, above the graduation threshold.

### Performance stratified by rural and urban settings

The mean percentage score in the human resource domain was statistically significantly higher in graduated than non-graduated districts in both rural and urban settings; 92.7% versus 55.5% in rural settings (OR = 10.23, 95% CI: 6.49–16.12, P <0.001) and 94.1% versus 89.8% in urban settings (OR = 1.80, 95% CI: 1.13–2.89, P = 0.014), respectively (Table 4).

**Table 4:** Graduation Performance Stratified by Rural and Urban Setting.

		Rural				Urban			
Domain	Program Area	Graduated	Non-Graduated	OR (95% CI)	P-Value	Graduated	Non-Graduated	OR (95% CI)	P-Value
**Commodity management**	CT-PMTCT	93.8%	95.6%			98.8%	98.9%		
	Laboratory	92.3%	93.3%			93.4%	95.8%		
	Pharmacy	95.0%	96.4%			89.2%	87.2%		
**Mean percentage score**	** **	**94.0%**	**95.8%**	**0.69 (0.46–1.03)**	**0.067**	**92.9%**	**92.7%**	**1.02 (0.73–1.43)**	**0.904**
**Data management**	ART-CC	87.8%	89.9%			95.4%	95.6%		
	CT-PMTCT	97.4%	100.0%			98.6%	100.0%		
	Laboratory	89.3%	88.9%			100.0%	97.9%		
	Pharmacy	93.0%	89.6%			98.0%	95.7%		
**Mean percentage score**	** **	**95.9%**	**96.9%**	**0.77 (0.46–1.27)**	**0.305**	**98.0%**	**98.3%**	**0.86 (0.41–1.82)**	**0.691**
**Human resource management**	ART-CC	100.0%	95.8%			92.5%	90.1%		
	CT-PMTCT	99.3%	72.9%			99.1%	97.4%		
	Laboratory	73.3%	51.9%			92.8%	90.3%		
	Pharmacy	88.0%	31.3%			93.0%	85.3%		
**Mean percentage score**	** **	**92.7%**	**55.5%**	**10.23 (6.49–16.12)**	**<0.001**	**94.1%**	**89.8%**	**1.80 (1.13–2.89)**	**0.014**
**Technical Capacity**	ART-CC	91.0%	92.4%			91.8%	93.5%		
	CT-PMTCT	92.4%	92.7%			95.4%	95.8%		
	Laboratory	81.8%	83.3%			89.5%	89.0%		
	Pharmacy	83.5%	83.7%			91.9%	91.2%		
**Mean percentage score**	** **	**90.3%**	**90.0%**	**1.04 (0.86–1.26)**	**0.698**	**92.9%**	**93.2%**	**0.96 (0.76–1.21)**	**0.711**
**Overall mean percentage score**	** **	**92.4%**	**89.4%**	**1.43 (1.24–1.65)**	**<0.001**	**93.9%**	**93.6%**	**1.05 (0.88–1.24)**	**0.601**

The overall mean percentage score for all four domains was statistically significantly higher in graduated than non-graduated districts in rural settings; 92.4% versus 89.4% (OR = 1.43, 95% CI: 1.24–1.65, P <0.001). However, this finding was not statistically significant in urban settings (Table 4).

Except in commodity management domain, urban facilities in graduated districts had higher mean percentage scores than rural facilities in graduated districts in data management, human resources management and technical capacity domain. (Table 4).

### Performance stratified by province

**Fig 3:**
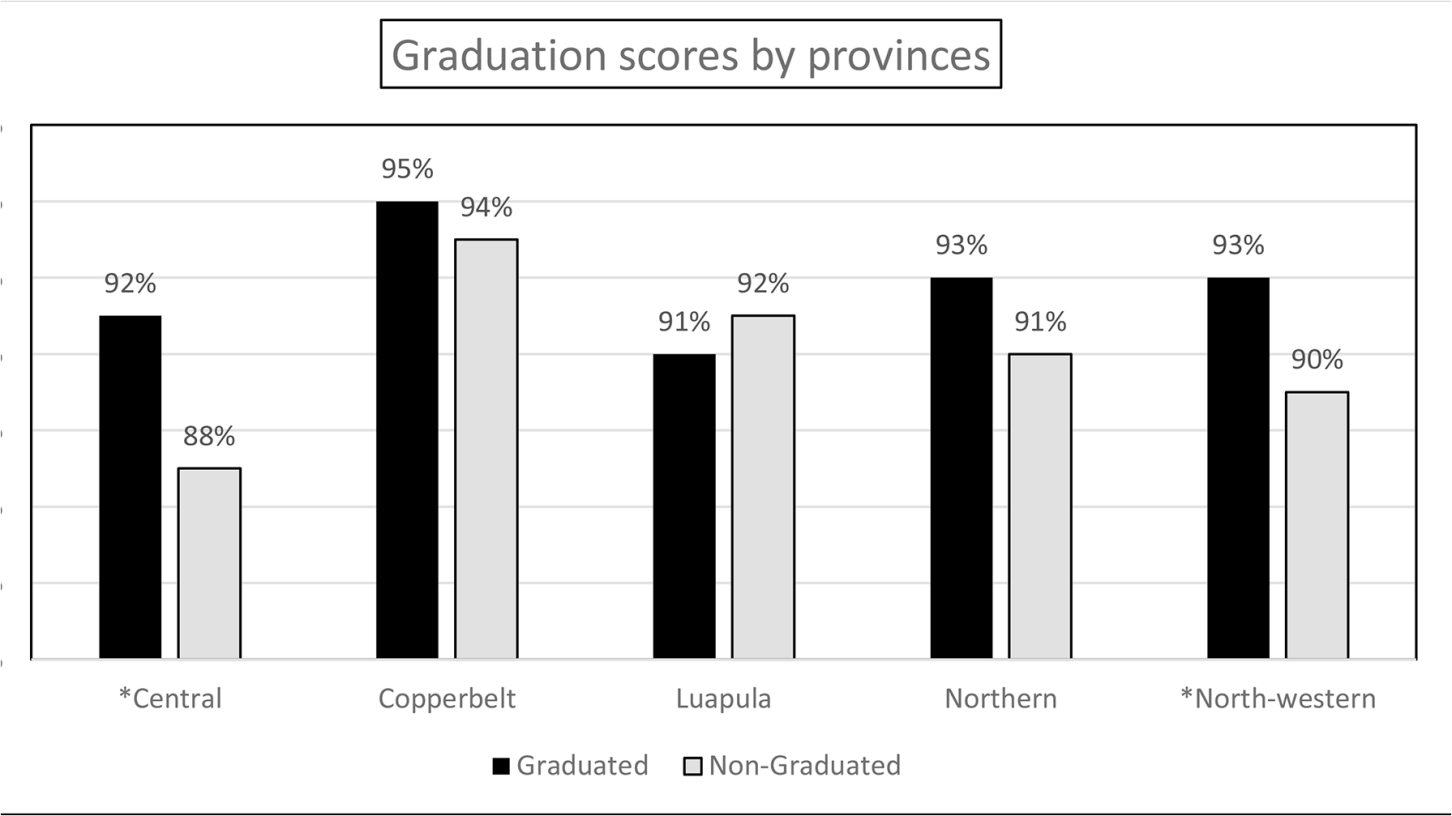
Graduation scores by province in graduated and non-graduated districts. Central OR 1.61 (95%CI: 1.31–1.97), P<0.001; Copperbelt OR 1.12 (95%CI: 0.90–1.39), P = 0.279; Luapula OR 0.87 (95%CI (0.66–1.15), P = 0.322; Northern OR 1.33 (95%CI (1.00–1.78), P = 0.052; North-western OR 1.45 (95%CI (1.07–1.96), P = 0.015.

The overall mean percentage score stratified by province was 90% and above in both graduated and non-graduated districts in four of the five provinces; the overall mean percentage score in Central province for districts that did not graduate was 88% (Fig 3). The overall percentage score was statistically significantly higher in graduated than non-graduated districts in Central province (OR = 1.6, 95% CI: 1.31–1.97, P <0.001) and North Western province (OR = 1.45, 95%CI: 1.07–1.96, P = 0.015).There was no statistically significant difference between graduated and non-graduated districts in the other provinces. In both Central and North Western provinces lack of human resource management contributed to this overall difference observed in graduated versus non-graduated districts; in Central province the mean percentage score for human resource management domain was 86.4% in graduated districts and 38.7% in non-graduated districts (OR = 10.03, 95%CI: 5.29–16.98, P <0.001), while in North-western province it was 91.8% in graduated districts and 61.2% in non-graduated districts (OR = 7.12, 95%CI: 3.17–16.00, P <0.001).

## Discussion

This study found that the overall mean percentage score for all four domains was statistically significantly higher in districts that graduated than districts that did not, indicating that it is feasible to use the QA/QI tool to measure service performance of health facilities and determine district graduation. The QA/QI tool can be used to establish the process required to support health facilities in attaining high levels of technical and management competency and progress toward independence from technical and programmatic assistance in low resource settings. The QA/QI process can be used in identifying performance gaps in health facilities that could be strengthened through collaboration between government and donors; where the donor progressively strengthens the gaps while preparing government to take over the full support for HIV/AIDS activities.

This study found that human resource is an important factor in determining district graduation. Like most low-income countries, Zambia has a shortage of skilled health professionals [8]. This has a negative effect in the provision of quality health services including services for HIV/AIDS care. This was demonstrated in our study where we found that the contributing factor to low performance in the human resource domain in non-graduated districts was not having at least two staff trained in pharmaceutical management of commodities for ART and opportunities infection, use of the anti-retroviral drugs logistics system, and general logistics management information system, as measured by the QA/QI tool. Even in rural and urban setting separately, the human resource management domain was still an important factor in determining district graduation. This was also observed in Central and North Western provinces where lack of human resource management contributed to the overall mean percentage score that was statistically significantly higher in graduated and non-graduated districts.

We found that in the rural setting, the overall mean percentage score for all four domains was statistically significantly higher in graduated than non-graduated districts. This suggests that the more rural health facilities there are in a district, the less likely that district will graduate. The lack of statistically significant difference in the overall mean percentage score between graduated and non-graduated districts in the urban settings could be because urban based health facilities have more and better medical equipment and human resources which could result in most facilities meeting graduation criteria. Unlike urban areas, rural settings have the lowest number of health workers, compared to urban areas [9]. Therefore, there is need for more effort in districts that have a greater proportion of rural rather than urban health facilities so that they are able to meet the graduation standards. However, in our study there seemed to be an indication that the human resource management domain in ART-CC services in rural settings performed better than in urban settings in both graduated and non-graduated districts. This could be due to strong community involvement in ART-CC services that exists in rural areas through volunteers that work as adherence support workers and lay counsellors. Regardless of whether the health facility is in a rural or urban settings, ZPCT II offered small monetary support to adherence support workers and lay counsellors found in these health facilities However, because of the cost of living which is lower in rural areas, this support is an incentive which translates in volunteers having a profound effect on HIV/AIDS service delivery in rural areas.

The other findings are that there was high performance, above the graduation threshold, in the commodity management domain in both graduated and non-graduated districts. This could be due to the Zambian government’s efforts, with support from USAID through John Snow Incorporation that improved distribution of essential pharmaceuticals that started in 2009 in these districts. Furthermore, a “bottom-up” quantification method for pharmaceuticals was introduced by the Zambian government, which provided necessary information for forecasting and avoiding drug stock outs [8].

We also found high performance, above the graduation threshold, in data management in both graduated and non-graduated districts. This could be attributed to the support that ZPCT II offered to MOH in paying salaries for data entry clerks (DECs). The DECs collected and managed routine data in HIV services for the MOH, in health facilities that offered ART. ZPCT II also provided mentorship to health workers in the health facilities on data management and reporting. Since the introduction of DECs in 2006, the timeliness and quality of data have improved greatly [10]. Government is now considering including DECs in their district health establishments. To further improve data quality, the DECs visited other health facilities in the district that did not offer ART; where they took one day in a month to also collect data and compile reports that were sent to MOH and ZPCT II.

We found low performance in Laboratory and Pharmacy services in the technical capacity domain in both graduated and non-graduated districts. The low performance in Laboratory services was due to lack of adequate storage space for laboratory reagents, inadequate timely external referral systems for laboratory specimen and non-participation in external quality assessment scheme for CD4, as measured by the QA/QI tool. The low performance in Pharmacy was due to inappropriate maintenance of storage conditions in the dispensing area, lack of adherence to good dispensing practices when dispensing drugs to patients, and lack of and non-use of appropriate standard operating procedures/guidelines, as also measured by the QA/QI tool.These weaknesses in Laboratory and Pharmacy services were also observed in the Zambia National Health Strategic Plan for 2011–2015 [8]. The low performance in Laboratory and Pharmacy technical areas is also linked to the human resource management domain as observed in our study. This could be due to the nature of work in these two technical areas that require specific skills that are not easily transferable compared to ART-CC and CT-PMTCT; where task shifting is common. Task shifting is better implemented within ART-CC and CT-PMTCT services than in Laboratory and Pharmacy services. Nurses are now undertaking a range of tasks that were formerly the responsibility of doctors, while certain tasks that were for nurses have been shifted to adherence support workers and lay counsellors [10].

This study had the following limitations; the nature of data used was for routine QA/QI assessments which only measured four performance domains. However, there could be other domains we did not measure that include management capacity (leadership and governance) and health financing systems of the districts [11]. The study gave equal weight to all the four performance domains when in reality some domains could have been more important than others [12]. The strength of this study is that most of the ZPCT II-supported health facilities and their respective districts were assessed for graduation and all those that were assessed were included in this study. Also, the QA/QI tool that was used to measure district service performance and graduation was based on MOH national guidelines and standard operating procedures.

## Conclusions

This study has shown that it is feasible to use a QA/QI tool to assess performance of health facilities and determine district graduation. The human resources management domain was found to be an important factor associated with district graduation. This association with district graduation was also shown to be important in rural and urban settings separately. Furthermore, the human resource domain also contributed to performances seen in all technical service areas. In both graduated and non-graduated districts, the performance in the other three domains (commodity management, data management and technical capacity) was above the graduation threshold which could have been as a result of the extra effort that the donors provided to government through payment of DECs for data management; improved distribution of essential pharmaceuticals in commodity management domain, and the support of task-shifting through payment of adherence support workers and lay counsellors under the technical capacity domain. Health system strengthening interventions should therefore take into account human resources challenges in resource limited settings and should be based on empirical evidence on what donors can do in preparing government to take over management of health systems.
